# Identification of Diagnostic Metabolic Signatures in Thyroid Tumors Using Mass Spectrometry Imaging

**DOI:** 10.3390/molecules28155791

**Published:** 2023-07-31

**Authors:** Xinxin Mao, Luojiao Huang, Tiegang Li, Zeper Abliz, Jiuming He, Jie Chen

**Affiliations:** 1 Department of Pathology, Peking Union Medical College Hospital, Chinese Academy of Medical Sciences and Peking Union Medical College, Beijing 100730, China; maoxinxin@pumch.cn; 2 State Key Laboratory of Bioactive Substance and Function of Natural Medicines, Institute of Materia Medica, Chinese Academy of Medical Sciences and Peking Union Medical College, Beijing 100050, China; luojiao.huang@maastrichtuniversity.nl (L.H.); litiegang@imm.ac.cn (T.L.); zeper@imm.ac.cn (Z.A.)

**Keywords:** thyroid follicular adenoma, thyroid follicular carcinoma, mass spectrometry

## Abstract

“Gray zone” thyroid follicular tumors are difficult to diagnose, especially when distinguishing between benign follicular thyroid adenoma (FTA) and malignant carcinoma (FTC). Thus, proper classification of thyroid follicular diseases may improve clinical prognosis. In this study, the diagnostic performance of metabolite enzymes was evaluated using imaging mass spectrometry to distinguish FTA from FTC and determine the association between metabolite enzyme expression with thyroid follicular borderline tumor diagnosis. Air flow-assisted desorption electrospray ionization mass spectrometry imaging (AFAIDESI-MSI) was used to build a classification model for thyroid follicular tumor characteristics among 24 samples. We analyzed metabolic enzyme marker expression in an independent validation set of 133 cases and further evaluated the potential biological behavior of 19 thyroid borderline lesions. Phospholipids and fatty acids (FAs) were more abundant in FTA than FTC (*p* < 0.001). The metabolic enzyme panel, which included FA synthase and Ca^2+^-independent PLA2, was further validated in follicular thyroid tumors. The marker combination showed optimal performance in the validation group (area under the ROC, sensitivity, and specificity: 73.6%, 82.1%, and 60.6%, respectively). The findings indicate that AFAIDESI-MSI, in combination with low metabolic enzyme expression, could play a role in the diagnosis of thyroid follicular borderline tumors for strict follow-up.

## 1. Introduction

Thyroid tumors are the most common endocrine lesions worldwide, and their incidence has continuously increased over the last three decades [1,2]. Different clinical examinations, including ultrasound, CT, MRI and fine-needle aspiration cytology, have been utilized for the preoperative diagnosis of thyroid tumors. Of these examinations, pathology remains the gold standard for clinical diagnosis, and thyroid tumors arising from follicular cells can generally be categorized into malignant and benign tumors according to their pathology. However, it is occasionally extremely challenging to differentiate thyroid follicular adenoma (FTA) from follicular thyroid carcinoma (FTC). In these cases, pathomorphology is used to describe the characteristics of malignant tumors, named “atypia,” which do not appear in benign tumors. However, this morphological standard does not apply to thyroid follicular neoplasms. FTA cells can exhibit atypia, whereas FTC cells may show minimal or absent atypia. Moreover, distant metastasis of FTC sometimes occurs, such as in bones or lungs, and the morphology of metastatic tumors resembles that of FTA. Currently, FTC diagnosis is defined by capsular and/or vascular invasion; however, this standard is difficult to achieve in frozen sections or even surgically excised lesions. 

As a result, in the fifth version of the World Health Organization (WHO) classification, follicular borderline lesions, which include non-invasive follicular thyroid neoplasms with papillary-like nuclear features (NIFTP), and thyroid tumors of uncertain malignant potential (UMP) have been proposed to classify follicular thyroid neoplasms with questionable capsular or vascular invasion. The prognosis of benign FTA is excellent and only requires surgical resection of the tumor. In contrast, the treatment of malignant FTC requires bilateral thyroidectomy and lymph node dissection, combination therapy with I131, and the administration of thyroxine tablets for life. Surgeons hope that a pathological diagnosis, especially an intraoperative frozen diagnosis, can provide accurate and timely diagnostic information to guide clinical operations and postoperative treatment, but discriminating between FTA and FTC is always the greatest clinical challenge for pathologists. Therefore, the proper classification of thyroid follicular diseases has become an urgent clinical need.

Mass spectrometry imaging (MSI) is an efficient method applied to tumor research, allowing the identification of different classes of molecular species in tumor surgical margin studies, tumor qualitative diagnoses, the prediction of cancer lymph node metastasis, and prognostic assessments of the neoplasm [3,4,5,6,7]. The major advantage of MSI is the ability to combine molecular information and histomorphological images. The obtained molecules can have high tissue specificity and are closely related to clinical information, presenting clear information on molecular organization [8,9]. Moreover, MSI technology can provide effective auxiliary diagnostic methods for pathological histology, meaning that medical experts will not only define tissue types based on the morphological structure of the tissue, but can also include their molecular components. Metabolomic research is closely related to the occurrence and development of tumors, and changes in the expression of metabolite molecules and related metabolic pathways are important characteristics of tumor cells. Moreover, metabolomic research based on MSI is a high-throughput approach that can identify molecular information closely related to the occurrence and development of tumors. 

The airflow-assisted desorption electrospray ionization (AFAIDESI)-MSI technique under ambient conditions is a high-resolution ambient molecular imaging method. Here, we employ AFAIDESI-MSI metabolomic analysis to define novel diagnostic pathways and metabolites for thyroid follicular tumors, ultimately serving as potential markers of malignant tumors with uncertain potential. Our prospective study aimed to develop and validate an AFAIDESI-MSI metabolomic analysis to define novel diagnostic pathways and metabolites to discriminate between FTA and FTC and improve the diagnosis of indeterminate cases. 

## 2. Results

### 2.1. Thyroid Tumor Tissue Characteristics Determined Using AFAIDESI-MS Imaging and Tumor-Associated Metabolic Pathways

**Figure 1:**
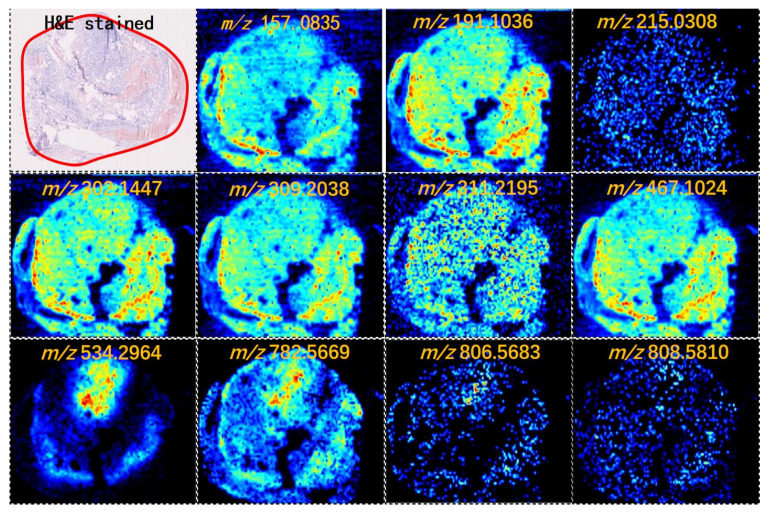
Mass spectrometry (MS) images of representative metabolites in thyroid tumors (intensity of the color scale represents the relative value). H&E, hematoxylin and eosin.

**Figure 2:**
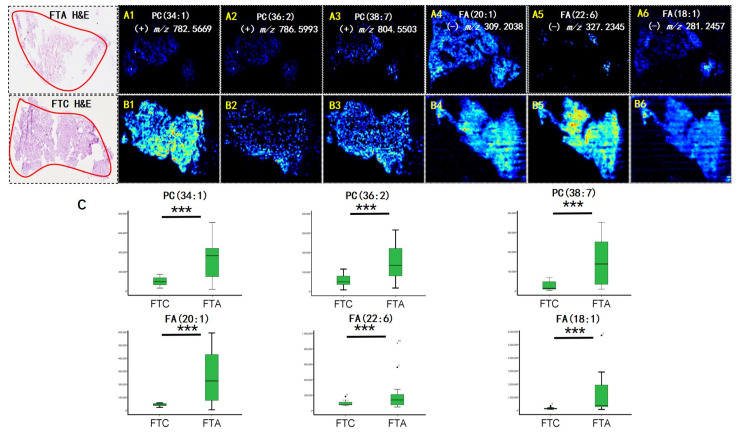
Air flow-assisted desorption electrospray ionization mass spectrometry imaging (AFAIDESI-MSI) of follicular thyroid adenoma (FTA) and follicular thyroid carcinoma (FTC). The distribution of (**A1**,**B1**) *m*/*z* 782.5669, PC (34:1); (**A2**,**B2**) *m*/*z* 786.5993, PC (36:2); (**A3**,**B3**) *m*/*z* 804.5503, PC (38:7); (**A4**,**B4**) *m*/*z* 309.2038, FA (20:1); (**A5**,**B5**) *m*/*z* 327.2345, FA (22:6); (**A6**,**B6**) *m*/*z* 281.2457, FA (18:1). (**C**) The statistical box plots display the intensity of metabolites. *** *p* < 0.001. H&E, hematoxylin and eosin.

AFAIDESI-MSI was performed using negative and positive ion modes on 24 thyroid tumor tissue samples comprising 5 FTC and 19 FTA. Figure 1 shows the MS images of the representative thyroid tumor metabolites. We built a classification model that could distinguish between FTC and FTA tumors. Multivariate statistical methods for the training subset included principal component analysis (PCA) and orthogonal partial least squares discriminant analysis (OPLS-DA). A supervised PCA was initially performed to obtain an overview of all tumors. The differences between the FTA and FTC were explored using OPLS-DA. Threefold cross-validation was performed on a pixel-by-pixel basis in the positive ion mode using the 912 peaks evaluated in the positive mode. The classification of the FTA and FTC groups resulted in 1 predictive (tB pB) and 3 orthogonal (tB oB) (1 + 3) components, with a cross-validated predictive ability, Q2 (cum), of 73.3%. Additionally, 67.9% of the variance in R2 (X) accounted for 86.9% of the variance in R2 (Y), as shown in Supplementary Figure S1A. Phosphoric acid species, including phosphatidylcholine (PC) (34:1), PC (36:2), and PC (38:7), were among the selected features with the greatest weight for characterizing thyroid tumors (Figure 2A). Negative ion mode data were analyzed to predict FTC and FTA. Following the same strategy in the negative mode, an overall agreement of 74.2% resulted in 1 predictive (tB pB) and 13 orthogonal (tB oB) (1 + 4) components with a cross-validated predictive ability, Q 2 (cum) of 74.2%, and 72.2% of the variance in R 2 (X) accounted for 92.4% of the variance of R2 (Y) (Supplementary Figure S1B). Among the selected features, several fatty acids (FAs), including FA 20:1 (*m*/*z* 309.2038), FA 18:1 (*m*/*z* 281.2457), and FA 22:6 (*m*/*z* 327.2345) were detected (Figure 2B). Interestingly, all ions exhibited a higher abundance in FTA than in FTC.

Changes in the expression of metabolic enzymes and related pathways are critical characteristics of tumorigenesis. Metabolic enzymes associated with complex metabolic reactions act as key nodes in biological metabolic networks and have always been recognized as potential diagnostic markers. AFAIDESI-MSI data combined with OPLS-DA analysis enabled the determination of region-specific metabolites. Subsequently, the discriminating metabolites were imported into the Kyoto Encyclopedia of Genes and Genomes (www.kegg.jp, accessed on 28 January 2023) to perform metabolic pathway matching analysis, which facilitated the discovery of altered metabolic pathways. The findings indicate that PC metabolism and FA biosynthesis are considerably dysregulated in thyroid tumors. Notably, two crucial metabolic enzymes were directly associated with alterations in identified metabolic enzymes; specifically, fatty acid synthase (FASN) and calcium-independent phospholipase A2 (iPLA2) catalyzed the formation of long-chain FAs and biosynthesis of PC, respectively.

### 2.2. The Protein Expression Levels of FASN and iPLAs Were Significantly Upregulated in Thyroid Adenoma

We assessed the classifier’s performance identified using immunohistochemical (IHC) staining on an independent set to further evaluate the metabolite enzyme validation. This group included 113 thyroid tumors, comprising 28 FTC and 66 FTA. The FTC group comprised 6 males and 22 females. Furthermore, the mean age was 47.3 years (ranging: 20–69 years). The FTA group comprised 6 males and 60 females. The mean age was 43.75 years (range: 25–74 years). 

**Figure 3:**
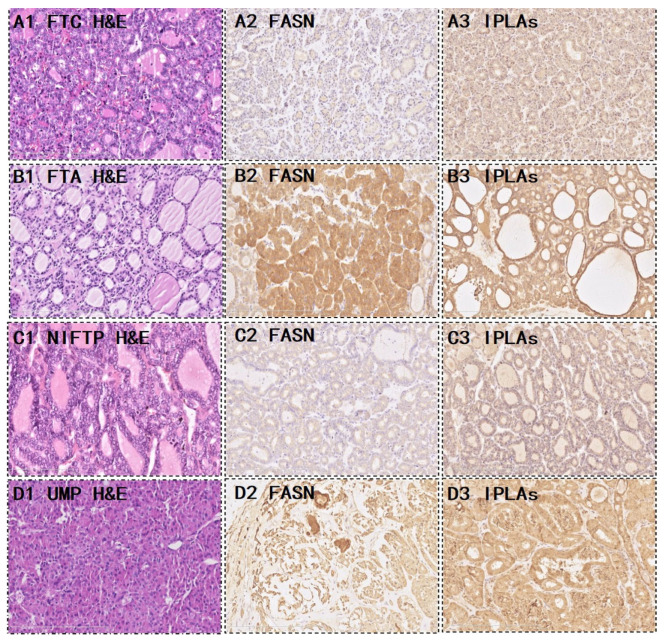
Representative morphological and expression of the two metabolic enzymes. FTC (**A**) and FTA (**B**); Hematoxylin and eosin (H&E) staining for (**A1**) FTC, (**A2**) FASN, and (**A3**) iPLAs, which showed weak or moderate cytoplasmic staining. H&E staining for (**B1**) FTA, (**B2**) FASN, and (**B3**) iPLAs showed strong positive staining. (**C**,**D**) show the metabolic enzyme expression in borderline thyroid tumors. (**C1**,**D1**) H&E stained for an NIFTP and a UMP, (**C2**,**D2**) showed FASN staining, and (**C3**,**D3**) showed iPLAs staining. NIFTP, non-invasive follicular thyroid neoplasm with papillary-like nuclear features; UMP, uncertain malignant potential.

**Figure 4:**
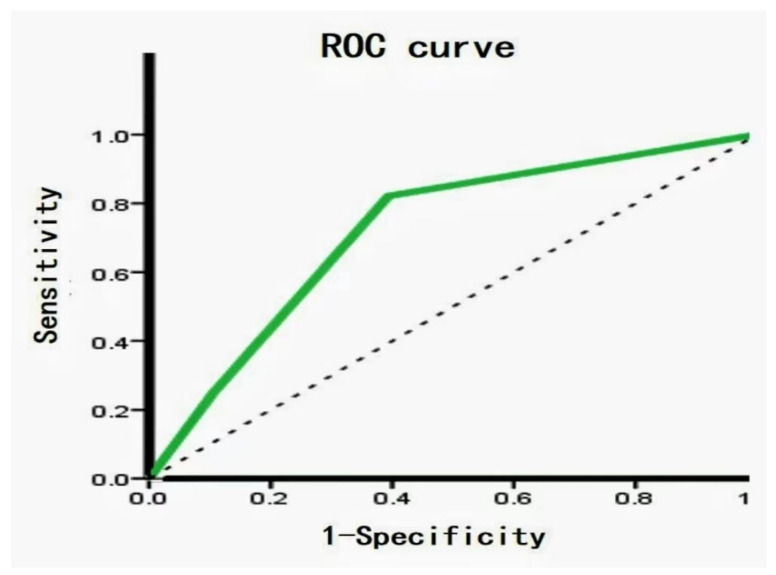
Receiver operating characteristic (ROC) analysis of metabolite biomarkers. ROC between FTA and FTC showed an area under the curve (AUC) value 73.6% (95% CI: 62.7–84.5%), with a sensitivity and specificity of 82.1% and 60.6%, respectively.

Out of the 28 FTC, 9 (32.1%) were strongly positive and 19 (67.9%) were weakly to moderately positive for FASN. Five (17.9%) iPLAs were strongly positive, 16 (57.1%) were weakly to moderately positive, and 7 (25%) were completely negative for FASN. Out of the 66 FTA, 37 (56.1%) were strongly positive for FASN, 28 (42.4%) were weakly to moderately positive, and one (1.5%) was completely negative. Furthermore, 40 (60.6%) iPLAs were strongly positive, 19 (28.8%) were weakly positive, and seven (10.6%) were negative. Interestingly, we noticed that the metabolic enzyme was significantly higher in the FTA than in the FTC (*p* = 0.043 and *p* = 0.0001 in FASN and iPLAs, respectively) (Table 1). Figure 3A,B shows the metabolic enzyme expression in FTC and FTA. The diagnostic potential of FASN and iPLAs was identified using receiver operating characteristic (ROC) curve analysis. The area under the curve (AUC), an accuracy index for evaluating the predicting performance, was 61.4% for FASN (95% confidence interval [CI]: 49.2–73.7%), compared with 71.9% for tissue iPLAs (95% CI: 60.9–83.0%). Subsequently, we combined the two biomarkers into a diagnostic combination; ROC curve analysis showed that the AUC was 73.6% (95% CI: 62.7–84.5%), with a sensitivity and specificity of 82.1% and 60.6%, respectively (Figure 4).

### 2.3. Novel Diagnostic Workflow Based on AFAIDESI-MSI Analysis and Metabolite Enzyme Markers

We proposed a novel workflow that combines histopathology, AFAIDESI-MSI analysis, and metabolite enzyme markers to diagnose borderline thyroid tumors for implementation in clinical applications. Here is a demonstration of the performance of the combined metabolic enzyme markers and predictive model. In the 19 settings, 5 NIFTP and 14 thyroid tumors of UMP were collected. Female patients accounted for the vast majority in all groups. The female:male ratio was 15:4. The mean age was 42.8 years. The median follow-up was 36.4 (range 11–90) months. From the immunohistochemistry results, 5 (26.3%) cases were more inclined to be FTC, and 12 (63.2%) cases were more likely to be FTA (Table 2). Figure 3C,D shows the metabolic enzyme expression in borderline thyroid tumors.

Two cases could not be classified according to the metabolite enzyme marker results because of inconsistent expression intensities of FASN and iPLAs; therefore, we entered the two cases of mass spectrometry datasets into the predictive model. The results showed that both the IMS datasets were in the FTA category. Additionally, we randomly selected three borderline cases of MSI data for predictive model analysis; the results of IMS were consistent with the results of metabolite enzyme expression.

## 3. Discussion

“Gray zone” thyroid follicular tumors, whether benign or malignant, are difficult to diagnose. Therefore, an accurate evaluation of resected benign or malignant thyroid follicular tumors after surgery is critical to avoid secondary surgery and over-diagnoses. This study is the first IMS analysis performed on UMP follicular tumors. Traditional cytologic specimens cannot be used to distinguish benign from malignant follicular neoplasms because the diagnosis of malignancy in follicular neoplasms requires tumor cells to present capsular or vascular invasion. However, both FTC and FTA are follicular thyroid lesions that cause challenging pathological issues. To date, pathologists are virtually unable to draw a clear distinction between FTC and FTA based on a frozen section examination alone. They have some overlapping morphological features; therefore, they represent a diagnostic dilemma for practicing pathologists. A well-known reliable pathological criterion for the malignant diagnosis of FTC is the presence of tumor cells invading the tumor capsule or blood vessels. However, the limited sample collection during the frozen diagnosis process makes evaluating all samples’ capsules and blood vessels difficult. Although no envelope or vascular infiltration was found in the paraffin specimen after a comprehensive evaluation, if substantial structural and morphological heterogeneity was observed, pathologists could not completely rule out the possibility of malignant tumor biology or provide clinically effective diagnosis and treatment guidance. Therefore, the recent WHO classification has proposed certain borderline lesions, for example, NIFTPs and UMP thyroid tumors. Nevertheless, proper classification of thyroid follicular diseases could be improved with better evaluation of clinical prognosis.

As a result, more reliable diagnostic markers are required. To date, there have been an increasing number of markers in the process of continuous evaluation of their diagnostic utility. For example, Kaliszewski et al. [10] proposed that serum thyroid-stimulating hormone levels are considerably higher in patients with atypia and follicular lesions of undetermined significance. Chuang et al. [11] suggested that IHC marker panels, including CK19, CD56, galectin-3, and some other antibody markers, can differentiate thyroid follicular neoplasms. Nevertheless, none of these biomarkers are routinely used because they lack validity.

MSI has been proven to be useful for molecular diagnoses and relies on professional imaging software. AFAIDESI-MSI is a high-resolution ambient molecular imaging method, and metabolomics research based on MSI can detect metabolic abnormalities in tumors with high throughput. In this study, MSI data combined with OPLS-DA analyses, as a powerful approach, was not only used to discriminate thyroid tumors from adjacent normal thyroid tissues, but also for tumor-specific discrimination. We can effectively mine spatial metabolite molecular information in human tumor tissue samples using high-resolution recognition and data processing methods to analyze the distribution of tumor molecules, thereby providing a better understanding of the metabolic heterogeneity of tumor tissue. The expression of phosphoric acid species and FAs was higher in the FTA group, and targeted IHC staining of the potential metabolic enzymes—including FASN and iPLAs—was performed on adjacent sections to validate our discovery. The internal validation of the classification model showed very good performance (95% CI: 62.7–84.5%, AUC = 73.6%, sensitivity = 82.1%, specificity = 60.6%). 

FAs are an essential component of cell membranes, playing a major role in maintaining the basic morphology of cells and their normal physiological functions. Multiple tumors and their early lesions undergo endogenous FA biosynthesis independent of extracellular lipid levels [12,13,14]. In this study, elevated FA levels in the tumor tissue were further confirmed by our MSI results. Figure 1 shows that the FA ion intensities demonstrated an increasing trend from the normal thyroid follicular epithelium to the pathological thyroid tumor epithelia. Interestingly, the ion intensity of FAs in FTA was higher than that in FTC (*p* < 0.001, Figure 2C), indicating that FAs may predict potential diagnoses in thyroid follicular tissues. Tumor cells rapidly synthesize FAs to meet energy consumption [15]. A multifunctional homodimeric FASN catalyzes the biosynthesis of endogenously synthesized FAs [16,17,18]. Therefore, FASN is the crucial metabolic regulatory enzyme responsible for the terminal catalytic step in FA synthesis. The adjacent IHC stain showed that FASN was primarily expressed in both FTA and FTC, and that its expression was higher in FTA. Some studies have indicated that early upregulation of FASN in precursor lesions may represent an obligatory metabolic acquisition in response to the microenvironment of preinvasive lesions, which continues to occur in malignant stages. The functional and temporal linkage between the glycolytic switch and FASN-related lipogenic phenotype may represent coevolved essential components of the malignant phenotype, signifying the hallmarks of invasive cancers [19].

Phospholipids are crucial components of the cell membrane. PC is a phospholipid that comprises the main components of cell membranes. The continuous de novo synthesis PC synthesis pathway provides tumor cells with components of synthetic cell membrane, lipid signal transduction, and energy for rapid growth of tumor cells [20]. The MS images indicated that phospholipids were significantly upregulated in the FTA tumor region compared with the FTC epithelium (*p* < 0.001, Figure 2C). Over the years, the metabolism of phospholipids and their metabolic products in mediating cell function have received considerable attention. Emerging studies have focused on iPLAs enzymes that mediate growth and signaling in numerous cell types. They may offer unique targets for treating various pathologies whose etiology involves the generation of phospholipid signals [21]. Some studies have demonstrated that iPLAs mediate cell growth by participating in signal transduction pathways, including epidermal growth factor receptors, mitogen-activated protein kinases, tumor suppressor protein p53, and cell cycle regulator p21 [22,23,24,25,26]. We speculate that the upregulated expression of iPLAs in thyroid follicular tumors may be related to elevated phospholipid biosynthesis. Similarly, IHC staining displayed that iPLAs expression was considerably higher in FTA. 

Subsequently, we focused on 19 patients with indeterminate pathological classifications. A prediction of benign diagnosis was obtained from 12 of the 19 patients that agreed with the follow-up, and 5 with a suspicious diagnosis were malignant. Due to the inconsistent expression intensity of FASN and iPLAs, two cases could not be classified according to the metabolite enzyme marker results. The two cases of mass spectrometry dataset predictive results were in the category of FTA. There are some explanations for this inconsistent expression, such as the heterogeneity of tumor cells or the instability that IHC antibodies may cause. As a result, this implementation of combined AFAIDESI-MSI with metabolite enzyme markers may positively affect the diagnosis classification for the “indeterminate” nodules.

## 4. Materials and Methods

### 4.1. Sample Collection

All postoperative thyroid tumor samples were collected at the Peking Union Medical College Hospital between 2014 and 2021. The Ethical Review Committee of the Peking Union Medical College Hospital approved the study protocols. All patients consented to participate in this study. Notably, none of the patients with thyroid cancer were administered preoperative treatment. All tumor tissue samples were stored at −80 °C until sectioning. Thereafter, 8-μm-thick tissue sections were used for the AFAIDESI-MSI experiments and 5-µm-thick tissue sections from adjacent slices were subjected to hematoxylin and eosin (H&E) staining to confirm the pathological diagnosis. In total, five sections were produced from each tumor tissue, four of which were used for mass spectrometry scanning using positive and negative ion modes, and one was stained with H&E before being placed on a slide box for long-term storage. Before the experiments, the slides were thawed at room temperature and then dried in a vacuum desiccator for approximately 1 h. 

### 4.2. AFAIDESI-MSI Analysis

The AFAIDESI-MS imaging analysis data were acquired in both positive and negative ion modes, changing one parameter value in a step-by-step manner and keeping the other parameters unchanged. The strength value of the mass spectrometry data obtained under the parameter setting was investigated, and the key parameters involved in the scanning process, including a spray solvent composition set at (±) 7000 V, spray solvent flow rate of 5 μL/min, and extraction flow rate of 45 L/min, were determined. The tissue surface and background portion of the tissue were used with a computer-controlled platform in the x direction at 200 μm until the entire tissue sample was completely scanned. The mass spectra were recorded in the full scan range of *m*/*z* (mass-to-charge ratio) 100–1000.

### 4.3. Histopathology Analysis

All thyroid tumor specimens were fixed in 10% buffered neutral formalin and embedded in paraffin. Samples were stained with hematoxylin for 2–3 min and washed with water for 5–10 s. Thereafter, they were subjected to 1% hydrochloric acid alcohol differentiation for 1–3 s and washed with water for 1 min. Next, they were subjected to reverse blue staining with 0.2% ammonia water for 3 s and washed with water for 1 min. Finally, staining with 1% eosin solution was performed for 2 min, with subsequent washing with water for 10 s. H&E-stained slides were used for re-diagnosis by two professional pathology professors, and they were classified according to the fifth version of the WHO criteria. The two pathologists determined the tumor content, and there was no necrosis or degeneration in any tissues. 

### 4.4. Immunohistological Staining

The expression of metabolic enzymes in thyroid tumor tissues was assessed via IHC staining using specific antibodies. Successive frozen tissue sections adjacent to the section analyzed using AFAIDESI-MSI were warmed to room temperature for 20 min. Sections were fixed in paraformaldehyde for 10 min. After washing in phosphate-buffered saline, the sections were immersed in 0.25% Triton X-100 for 15 min to make the tissue permeable, and then blocked with 1% bovine serum albumin for 30 min at room temperature. Furthermore, the sections were incubated with antibodies against iPLAs (Proteintech; Chicago, IL, USA; 22030-1-AP; 1:200) and FASN (Abcam; Toronto, ON, Canada, C; ab128870; 1:300) at 4 °C overnight, followed by rewarming at room temperature for 20 min. A PV-9000 two-step IHC kit was used according to the manufacturer’s instructions, and a DAB kit was used to detect antigen–antibody binding (Zhongshan Goldenbridge Biotechnology Ltd. Co., Beijing, China). The slides were counterstained with hematoxylin, dehydrated, mounted, and covered. FASN and iPLAs staining revealed cytoplasmic protein expression. The intensities of FASN and iPLAs were graded semi-quantitatively based on a scale comprising 0 (no staining), low expression (weak-to-moderate staining), and high expression (strong staining).

### 4.5. Data Processing and Statistical Analysis

The File converter function in Xcalibur was used to convert the series of raw data in .raw format, obtained via mass spectrometry scanning, into. cdf format. Thereafter, the actual length and width of the slice were inputted into the data import window of MassImager, and the raw data were imported in .cdf format in batches at once to generate the import sequence. The mass spectrometry peak detection slope was set to 100, the minimum peak intensity was set to 0, and the intensity integration method was set to total intensity. The calculation method for the mass-to-charge ratio was the weighted mass-to-charge ratio, and the in situ image of tissue slices covering the entire range of the mass-to-charge ratio (100–1000) was ultimately obtained. The endogenous metabolites that could represent the entire tissue profile were selected from the collected AFAIDESI-MSI mass spectrum profile, inputted into the ion channel corresponding to the mass–charge ratio in MassImager, and the distribution of this ion in tissue slices was obtained. Any background area outside of the tissue slice was obtained, the mass-to-charge ratio tolerance was set to 0.005, and background ions were deducted to obtain better imaging results. The H&E staining map of the tissue slices was imported; scaling, rotation, displacement, and other operations were performed in sequence; and they were then overlayed with representative ion imaging maps to accurately select the region of interest and output the average mass spectrometry map of that region for subsequent multivariate statistical analysis. Data from the metastatic and non-metastatic specimens were merged using Markview software 1.2.1 (AB Sciex, Framingham, MA, USA), and then, multivariate statistical analysis was conducted using PCA and OPLS-DA model recognition methods with SIMCA-P software 14.0.1 (Umetrics AB, Umea, Sweden). Data control analysis was conducted between the metastatic and non-metastatic groups to identify molecular information related to tumor metastasis. The extracted statistically significant mass-to-charge ratio (*m*/*z*) was used to image each sample, mass spectrometry imaging maps of each sample were evaluated, and spatial distribution characteristics and patterns of each molecule were discovered. After classification, the variable importance was assessed using an independent *t*-test (Microsoft Office Excel 2010, Los Angeles, CA, USA). The statistical significance level was set to *p* < 0.01. Lipid species were compared with LIPID MAPS (http://www.lipidmaps.org/, accessed on 28 January 2023), Massbank (http://www.massbank.jp/, accessed on 28 January 2023), and HMDB (http://hmdb.ca/, accessed on 28 January 2023) databases. ROC curve analysis was performed to determine the roles of accuracy and specificity in the predictability of potential molecules. The AUC ranged from 0.5 to 1.0. Complete separation of the values based on the indicator was performed using scores above 0.75 or 75%

## 5. Conclusions

This study shows the putative role of AFAIDESI-MSI in routine clinical triage. The findings indicate that the combination of AFAIDESI-MSI and metabolite enzyme markers is a promising approach to advancing the diagnosis of thyroid follicular nodules. These findings indicate that AFAIDESI-MSI, in combination with low metabolic enzyme expression, could be used in the diagnosis of “gray zone”thyroid follicular tumors, as well as in routine clinical triage for strict follow-up.

## Figures and Tables

**Table 1:** Performance of the metabolic enzyme expression in FTA and FTC.

Metabolite Enzyme Markers	IHC	Expression Level	FTA (66)	FTC (28)	*p* Value
FASN	Positive	Strong Weak-moderate	37 (56.1%)	9 (32.1%)	0.043
			28 (42.2%)	19 (67.9%)	
	Negative		1 (1.5%)	0	
iPLAs	Positive	Strong Weak-moderate	40 (60.6%)	5 (17.9%)	0.0001
			19 (28.8%)	16 (57.1%)	
	Negative		7 (10.6%)	7 (25%)	

**Table 2:** Predicted diagnosis based on the metabolic enzymes of the 19 nodules with indeterminate thyroid tumors.

Histopathology	Predicted Diagnosis		
	Benign	Gray Zone	Malignant
NIFTP (5)	4	0	1
UMP (14)	8	2	4

## Data Availability

The data presented in this study are contained within the article and Supplementary Materials.

## Supplementary Materials

The following supporting information can be downloaded at: https://www.mdpi.com/article/10.3390/molecules28155791/s1, Figure S1: (A) OPLS-DA score plots based on positive AFAIDESI-MSI data from FTC and FTA, (B) OPLS-DA score plots based on negative AFAI-MSI data from FTC and FTA; Table S1: The discriminated meatbolites between FTA and FTC.

## Sample Availability

Samples of the metabolites standard substances are available from the authors.
