# Supplementary material for: Identification of Diagnostic Metabolic Signatures in Thyroid Tumors Using Mass Spectrometry Imaging
Source: Molecules. 2023 Jul 31;28(15):5791. doi: 10.3390/molecules28155791 (PMC10421042; doi:10.3390/molecules28155791)
Supplement: Supplementary file 1 [file molecules-28-05791-s001.zip › molecules-2451523-supplementary.pdf]

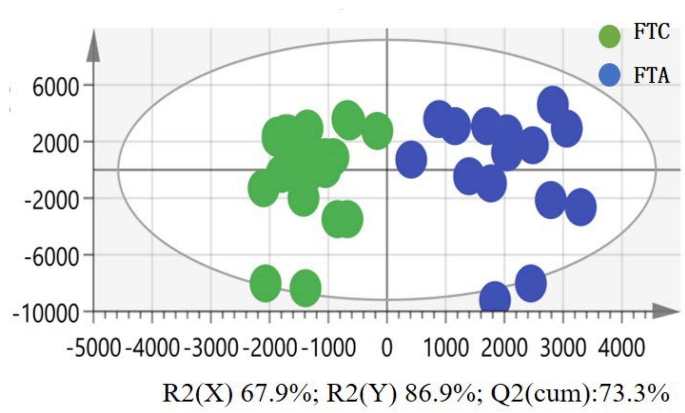

(**A**)

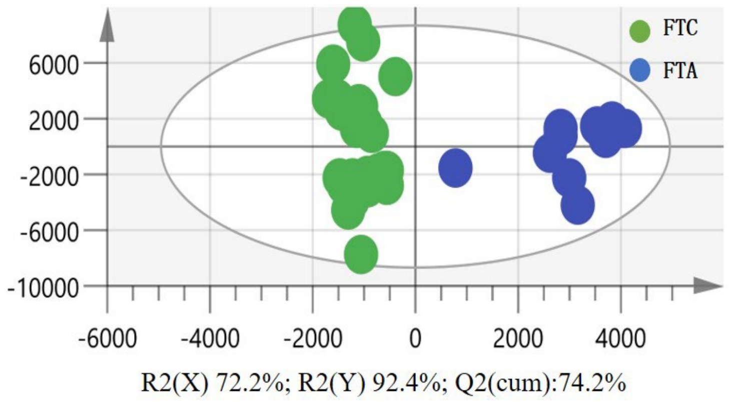

(**B**)

Figure S1. (**A**) OPLS-DA score plots based on positive AFAIDESI-MSI data from FTC and FTA.; (**B**) OPLS-DA score plots based on negative AFAI-MSI data from FTC and FTA.

Supplementary Table S1. The discriminated meatbolites between FTA and FTC.

Peak (*m/z*)	Formula	Identification	Ion type
758.5623	$C_{42}H_{80}NO_8P$	PC(34:2)	$[M+H]^+$
780.5435	$C_{42}H_{80}NO_8P$	PC(34:2)	$[M+Na]^+$
782.5669	$C_{42}H_{82}NO_8P$	PC(34:1)	$[M+Na]^+$
784.5823	$C_{42}H_{84}$ NO_8P	PC(34:0)	$[M+Na]^+$
786.5993	$C_{44}H_{84}NO_8P$	PC(36:2)	$[M+H]^+$
804.5503	$C_{46}H_{78}NO_8P$	PC(38:7)	$[M+H]^+$
806.5620	$C_{44}H_{82}NO_8P$	PC(36:3)	$[M+Na]^+$
808.581	$C_{44}H_{84}NO_8P$	PC(36:2)	$[M+Na]^+$
96.9677	H_3PO_4	Phosphoric acid	$[M-H]^-$
309.2038	$C_{20}H_{38}O_2$	FA(20:1)	$[M-H]^-$
283.2638	$C_{18}H_{36}O_2$	FA(18:0)	$[M-H]^-$
255.2321	$C_{16}H_{32}O_2$	FA(16:0)	$[M-H]^-$
329.2383	$C_{18}H_{34}O_5$	9,10,13-TriHOME	$[M-H]^-$
311.2195	$C_{18}H_{32}O_4$	9,10-DiHODE	$[M-H]^-$
215.0308	$C_6H_{12}O_6$	Glucose	$[M+Cl]^-$
281.2457	$C_{18}H_{34}O_2$	FA(18:1)	$[M-H]^-$
279.2291	$C_{18}H_{32}O_2$	FA(18:2)	$[M-H]^-$
121.0279	$C_7H_6O_2$	Hydroxybenzaldehyde	$[M-H]^-$
327.2345	$C_{22}H_{32}O_2$	FA(22:6)	$[M-H]^-$
293.2008	$C_{18}H_{30}O_3$	11-oxo-(7E,9E)-octdecadienoic acid	$[M-H]^-$
